# Computed Tomography-Guided Fine Needle Biopsies of Vertebral and Paravertebral Lesions in Small Animals

**DOI:** 10.3390/ani12131688

**Published:** 2022-06-30

**Authors:** Patricia Laborda-Vidal, Myriam Martín, Marc Orts-Porcar, Laura Vilalta, Antonio Melendez-Lazo, Alejandra García de Carellán, Carlos Ros

**Affiliations:** 1 Departamento de Medicina y Cirugía Animal, Facultad de Veterinaria, Universidad Cardenal Herrera-CEU, CEU Universities, Calle Tirant lo Blanch 7, 46115 Alfara del Patriarca, Valencia, Spain; 2 Department of Veterinary Clinical Sciences, Louisiana State University School of Veterinary Medicine, Skip Bertman Drive, Baton Rouge, LA 70803, USA; myriammarbe@gmail.com; 3 Pride Veterinary Centre, Riverside Road, Derby DE24 8HX, UK; orts.marc@gmail.com; 4 Servicio de Animales Exóticos, Hospital Veterinari Canis, 17006 Girona, Spain; lauravilalta84@hotmail.com; 5 Hospital Veterinario UCV, Universidad Católica de Valencia, Avenida Pérez Galdós 51, 46980 Valencia, Spain; melendezlazovcp@gmail.com (A.M.-L.); alejandra_vet@hotmail.com (A.G.d.C.); carlos.rosalemany@gmail.com (C.R.); 6 T-Cito, Avenida Pérez Galdós 51, 46980 Valencia, Spain; 7 Servicio de Anestesia y Unidad del Dolor, Memvet Centro de Referencia Veterinaria, 07003 Palma de Mallorca, Spain; 8 Servicio de Neurología, Memvet Centro de Referencia Veterinaria, 07003 Palma de Mallorca, Spain

**Keywords:** computed tomographic-guidance, fine needle biopsy, vertebral, small animals

## Abstract

**Simple Summary:**

Vertebral and paravertebral lesions are common in small animals. They usually have a deep localization or a difficult approach; therefore computed tomographic (CT)-guidance is suggested to overcome the difficulties and obtain samples to reach a diagnosis. The aim of this work was to describe the use of CT-guidance to obtain fine needle biopsies from vertebral and paravertebral lesions in small animals. The fine needle biopsies were taken from the vertebra, the intervertebral disc and the intervertebral foramen from 10 dogs and a ferret. Two infectious and nine neoplastic lesions were diagnosed. The procedure was successful in 91% of the cases. Cytology was diagnostic in 80% of the cases. The ratio of complications was 9%. Computed tomography-guided fine needle biopsy is a useful and safe technique for the diagnosis of vertebral and paravertebral lesions in small animals. However, a degree of expertise is important.

**Abstract:**

Fine needle biopsy (FNB) is an effective, minimally invasive and inexpensive diagnostic technique. Under computed tomography (CT)-guidance, lesions that have a difficult approach can be sampled to reach a diagnosis. The aim of this study is to describe the use of CT-guidance to obtain FNB from vertebral and paravertebral lesions in small animals. Ten dogs and one ferret that had undergone CT-guided FNB of vertebral and paravertebral lesions and had a cytological or a histological diagnosis were included in this retrospective study. The FNB samples were taken in four cases from the vertebra, in two cases from the intervertebral disc and in five cases from the intervertebral foramen. Two infectious and nine neoplastic lesions were diagnosed. The percentage of successful FNB was 91%. The percentage of samples with a cytological diagnosis was 80%. The percentage of complications was 9%. Limitations were the small number of animals in the study, the lacking complementary percutaneous biopsies for comparison, the lacking final histological diagnoses in some cases and the intervention of multiple operators. Computed tomography-guided FNB is a useful and safe technique for the diagnosis of vertebral and paravertebral lesions in small animals. However, a degree of expertise is important.

## 1. Introduction

Vertebral and paravertebral lesions are common in small animals. Often, a presumptive or differential diagnosis is made based on the clinical and neurological exam and the complementary tests, being diagnostic imaging exams the ones that usually provide the most relevant information. However, histopathologic or cytologic evaluation of a lesion is needed to reach a definitive diagnosis. The deep localization of these lesions and the frequently challenging approach makes ultrasound-guided sampling difficult or impossible [1]. The use of computed tomography (CT)-guidance to obtain a fine needle biopsy (FNB) or a tissue core biopsy (TCB) from vertebral and paravertebral lesions can overcome these difficulties and can contribute to the final diagnosis of these type of lesions.

The use of CT-guidance to obtain diagnostic samples from lesions was first described in 1994 in veterinary medicine [2,3]. Since then, several studies have been published in small animals using both stereotactic free-hand CT-guided techniques and CT-guidance devices [4,5,6,7,8,9,10,11,12]. Computed tomographic-guidance provides advantages in comparison to ultrasound-guided techniques, which are the most common sampling techniques in veterinary medicine. The main advantages are the lack of superimposition of structures, the high contrast resolution, the disregard for overlaying gas or bone and the superior visualization of the lesion, the needle and its pathway [3]. Therefore, sampling under CT-guidance is especially indicated for inaccessible or hardly accessible lesions under ultrasound-guidance, like intrathoracic lesions, intracranial lesions and vertebral and paravertebral lesions. There are several studies of CT-guided sampling of intrathoracic lesions [4,5,6], brain lesions [8,9,10,12] and bone lesions too [7].

The aims of this study are (1) to describe the use of free-hand CT-guided FNB taken from vertebral and paravertebral lesions, including vertebrae, intervertebral discs (IVD), spinal nerves and paraspinal musculature in small animals; (2) to calculate the percentage of satisfactory FNB procedures, the percentage of samples with a cytological diagnosis made from the FNB and the rate of complications. Based on the authors’ review of the literature, this is the first study in veterinary medicine describing the use of CT-guided sampling of vertebral and paravertebral lesions.

## 2. Materials and Methods:

### 2.1. Study Population

In this retrospective study, the medical records of small animals which underwent free-hand technique CT-guided FNB of vertebral and paravertebral lesions were reviewed. The study was performed at the Hospital Veterinario UCV during the period 2016 to 2021. Owner consent was obtained for any clinically indicated investigation on admission. Inclusion criteria were animals that had undergone a CT scan followed by a CT-guided FNB of suspected pathology in the vertebral or paravertebral region. Data retrieved from the medical records were: signalment, bloodwork, clinical and neurological exam, imaging findings, imaging diagnosis, location of the lesion, FNB diagnosis and final diagnosis, treatment and outcome/follow-up. If TCB (surgical or image-guided) or necropsy and histopathology was performed, biopsy results were recorded.

### 2.2. Equipment and Technique

Computed tomographic studies were performed using 16-slice CT scanner (Siemens SOMATOM Scope, Siemens Healthcare Diagnostics, Illinois, IL, USA). The most common protocol used consisted of a helical volumetric acquisition, using 1.5 mm collimation, pitch 1, 0.5 s rotation time, 130 kVp tube voltage and 150–200 mAs tube current. Reconstructions were more commonly generated with a 3 mm slice thickness using a standard (soft tissue) kernel and 0.75 mm slice thickness with a sharp (bone) kernel. Position of the animals was generally in dorsal recumbency for better quality of the images. Computed tomographic images were acquired prior to and after administration of intravenous iodinated contrast medium (Ultravist, iopromide, 300 mg I/mL, Bayer Pharma AG, 13342 Berlin, Germany) at a dose of 600 mg I/kg body weight. Contrast was injected manually or with a single head contrast pressure injector (Bayer Medrad Stellant) adjusting the injection rate to the weight of the animal (injection rate of 1.5–4 mL/s at maximum pressure limits of 300 lb/in). Computed tomographic studies were reviewed using an image analysis workstation using proprietary DICOM software (OsiriX Pixmeo, Geneva, Switzerland; V.4.1.1 64-bit). Window width and level were adjusted accordingly, and three-dimensional and multi-planar reformatting were used whenever necessary for optimal evaluation.

All animals were examined under general anaesthesia and monitored during the procedure. The more commonly used general anaesthesia protocol included premedication with medetomidine (0.002 mg/kg, intravenously) and butorphanol or methadone (0.2 mg/kg, intravenously), and induction with propofol or alfaxalone (titrated to effect, intravenously). After orotracheal intubation maintenance was with isoflurane (1.5–2 percent) in a mixture of oxygen and medical air via an appropriate breathing system. This protocol was varied slightly depending upon individual patient requirements. Monitoring included clinical assessment of anaesthetic depth, pulseoxymetry, electrocardiography, non-invasive blood pressure, inspired fraction of isoflurane and oxygen, rectal temperature and end tidal carbon dioxide. Spirometry loops were monitored in those patient’s that underwent mechanical ventilation.

Once the pre- and postcontrast CT studies were completed, acquisition was paused to permit assessment of the location and extent of the lesion and allow the selection of the target plane. Animals were repositioned in a way allowing easy access to the lesion (usually sternal recumbency) and were clipped in the area of interest with subsequent surgical preparation. Free-hand CT-guided percutaneous FNB were performed as has been previously described [3], except for the marks on the skin. A 22-gauge (GA) spinal needle (Quincke type point) and a 5 mL syringe were generally used. Usually, only one to two samples were taken from each lesion to reduce possible complications. The CT-guided samples were taken by a board-certified radiologist (PLV), a diagnostic imaging intern (MOP) or a board-certified neurologist (CR). Animals were monitored for complications during the following days.

The samples were examined by a board-certified clinical pathologist (AML) and stained with Modified-wright stain according to the Standard Operating Procedures.

### 2.3. Descriptive Analysis

The following categorical data were summarized in percentages: percentage of successful FNB procedures, percentage of samples with a cytological diagnoses and rate of complications. The percentage of successful FNB procedures was calculated as the ratio between the number of cases, in which the FNB needles could be successfully placed within the lesion with CT-guidance, and the total number of cases, expressed as a percentage. The percentage of samples in which a cytological diagnosis could be reached was calculated as the ratio between the positive diagnoses and the number of cases with a successful FNB procedure, expressed as a percentage. Some diagnoses were not confirmed with surgical biopsy, necropsy or follow up; therefore, accuracy could not be calculated. The ratio of complications was calculated as the number of animals that presented complications during or worsening after the FNB procedure from the total number of animals in the study, expressed as a percentage.

## 3. Results

The results of signalment, clinical and neurological exam, imaging findings, imaging diagnosis, location of the lesion, FNB diagnosis and final diagnosis of this study are summarized in Table 1. A total of 10 dogs of different breeds and one ferret were retrospectively recruited. The dogs included five intact males, four intact females, one neutered female, with a median age of 9 years (range 2–13 years). The ferret was a 6-year-old (geriatric) neutered female.

**Figure 1:**
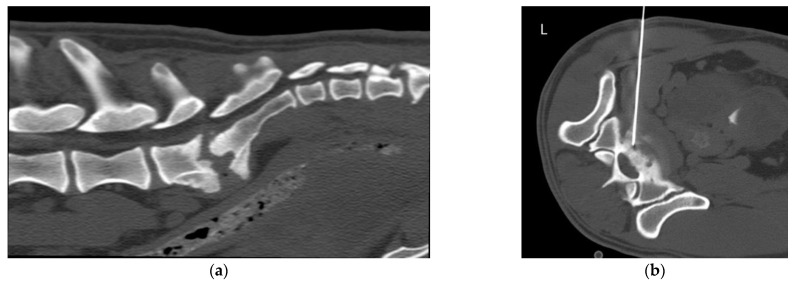
Case 2, 6-year-old Bullterrier with lumbosacral (LS) discospondylitis. (**a**) Sagittal multiplanar reconstruction (MPR) of the LS region in a bone window. There is moderate narrowing of the intervertebral disk space (IVDS) and severe osteolysis of the caudal L7 and cranial S1 endplates, with moderate sclerosis and marked spondylosis deformans; (**b**) Transverse MPR of the LS IVDS in a bone window. There is evidence of a spinal needle in the intervertebral space, introduced in a left ventrolateral approach.

**Figure 2:**
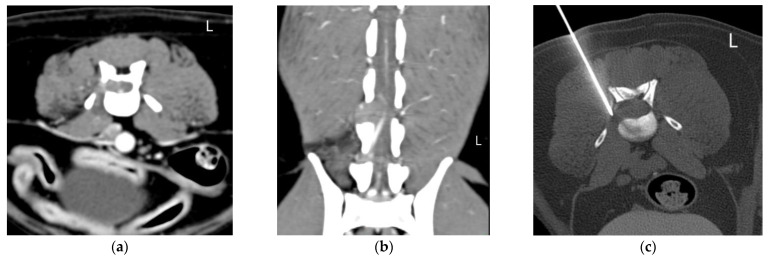
Case 3, 9-year-old Bodeguero andaluz with a L5–L6 right sided suspected peripheral nerve sheath tumor. (**a**) Transverse and (**b**) dorsal multiplanar reconstruction (MPR) of the lumbar spine (L5–L6) in a soft tissue window. There is an extramedullary intradural right-sided mass extending through the vertebral canal at the level of L5 and through the intervertebral foramen (IVF) at L5–L6. Note the muscle atrophy on the right epaxial muscles; (**c**) Transverse MPR at the level of the L5–L6 IVF in a bone window. There is evidence of a spinal needle in the mass in the IVF, introduced in a right dorsolateral approach.

**Figure 3:**
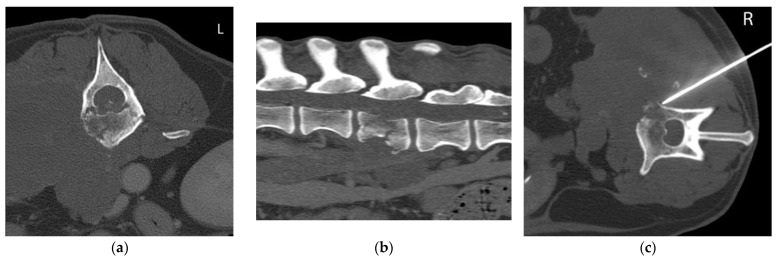
Case 6, 13-year-old Weimaraner with a polyostotic aggressive bone lesion (L3 and L4) associated to a hypaxial soft tissue mass consistent with a sarcoma. (**a**) Transverse and (**b**) sagittal multiplanar reconstruction (MPR) of the lumbar spine in a bone window. There is a large soft tissue mass in the right hypaxial muscles infiltrating the vertebral body and transverse processes of L3 and L4 and invading the retroperitoneal space. There is a pathological fracture of L3 vertebral body with vertebral compression; (**c**) Transverse MPR at the level of L3 in a bone window. There is evidence of a spinal needle in the hypaxial mass and the lytic L3 vertebral body, introduced in a right dorsolateral approach.

The CT-guided FNB samples were taken in 4 cases from vertebra (cases 1, 4, 6 and 11), in 2 cases from intervertebral disc (IVD) (cases 2 and 7) and in 5 cases from intervertebral foramen (IVF) (cases 3, 5, 8, 9 and 10). Examples from an IVD lesion, a spinal nerve lesion and a vertebral lesion and their respective FNB are shown in Figure 1, Figure 2 and Figure 3, respectively.

Attending to the region of the vertebral column where the FNB were taken, 6 FNB were taken from lesions in the lumbar column (cases 1, 3, 4, 5, 6 and 8), one FNB was taken from the lumbosacral IVD (case 2), two FNB were taken from lesions in the thoracic column (cases 7 and 10) and two FNB were taken from lesions in the cervical column (cases 9 and 11). The tip of the needle was observed within the lesion in nine cases and in the border of the lesion in one case (case 11). In one case (case 9), the FNB procedure was unsuccessful, and the lesion couldn’t be reached with the needle. Therefore, the percentage of successful FNB procedures was 91% (10/11). In the unsuccessful case, diagnosis was made based on necropsy and histopathology and it resulted a peripheral nerve sheath tumor.

The percentage of samples in which a cytological diagnosis could be reached from the FNB was 80% (8/10). In 6 cases, the diagnosis was confirmed either by surgical biopsy, by necropsy and histopathology or by clinical and therapeutic follow up. Two cases (20%) had an infectious etiology and eight cases (80%) were of neoplastic origin. Diagnosis was made in 2/10 cases based on microbiological culture and in 6/10 cases based on cytology of the CT-guided FNB samples. In 2/10 cases, FNB samples were not diagnostic, and diagnosis was made based on the surgical biopsy (cases 8 and 11). The final diagnoses were a peripheral nerve sheath tumor and an osteosarcoma, respectively. In case 2, a CT-guided percutaneous TCB was taken from the caudal endplate of L7 after the FNB procedure.

**Figure 4:**
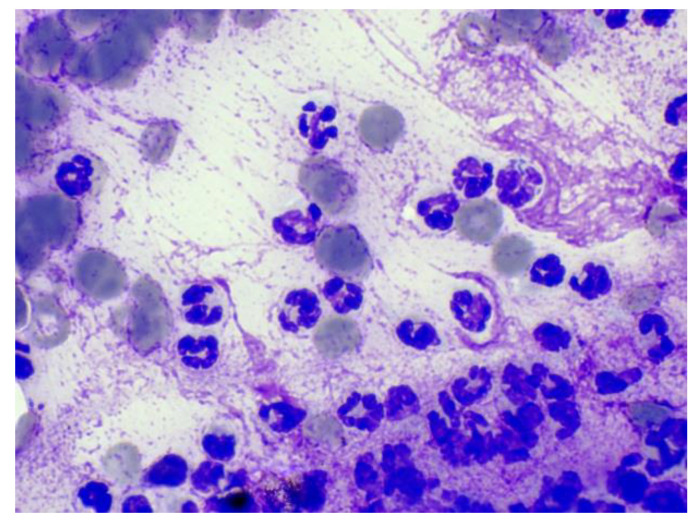
Degenerate neutrophils with intracellular bacilli consistent with suppurative septic discospondylitis (case 2). Modified-wright stain, 100×.

**Figure 5:**
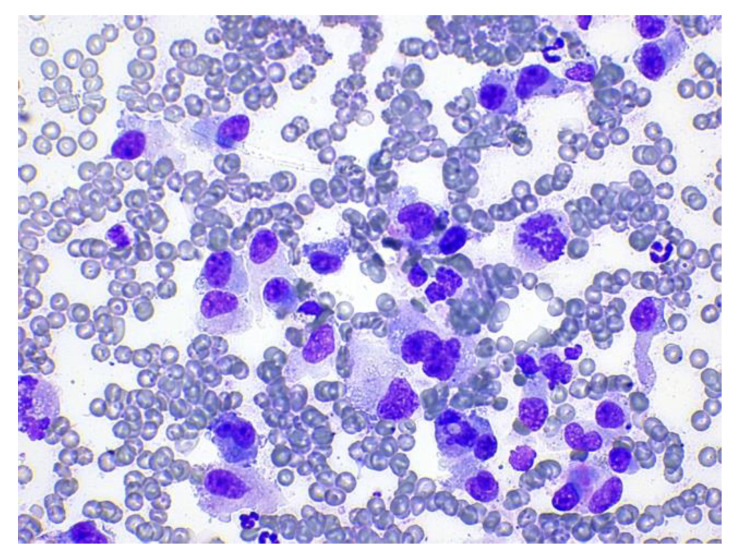
Mesenchymal cells with mild to moderate atypia consistent with sarcoma (case 6). Modified-wright stain, 40×.

Attending to the location and centering of the lesion, the lesions in the VB were classified as polyostotic aggressive bone lesions in two cases (cases 1 and 4; Table 1), monostotic aggressive bone lesion in one case (case 11) and infiltrating muscular and vertebral lesion in one case (case 6). In these cases, the diagnoses were: mesenchymal neoplasia consistent with sarcoma (cases 1 and 6), lymphoma (case 4) and osteosarcoma (case 11). In the two cases where the sample was taken from the IVD (cases 2 and 7), the final diagnosis was discospondylitis by *Enterobacter cloacae* and *Pseudomona aeruginosa*, respectively. In the cases where the sample was taken from the IVF (cases 3, 5, 8 and 10), the final diagnoses were mesenchymal neoplasia consistent with sarcoma (most likely peripheral nerve sheath tumor according to the neurological exam and the CT images) (cases 3, 8 and 10) and lymphoma (case 5). In cases 8 and 11, the samples were not diagnostic, as mentioned above. Figure 4 and Figure 5 are micrographs from cases 2 and 6, in which the cytological diagnoses were consistent with a septic discospondylitis and a sarcoma, respectively.

Most diagnoses were coincident with the suspected diagnosis made after the physical and neurological exam and after evaluating the CT images. No complications were observed immediately or during the following days of the minimally invasive procedure in 10 of the animals. Only one dog (case 2) (9%), the dog with lumbosacral discospondylitis, suffered worsening of the lameness and increased pain for one week after the procedure.

Follow up was possible in most of the cases: the cases with discospondylitis (cases 2 and 7) improved after treatment with appropriate/selected antibiotics. All cases with bone neoplasia (cases 1, 6, 7, 11), two cases with peripheral nerve sheath tumor (cases 3 and 9) and the case with the extradural infiltrative lymphoma (case 5) were euthanized immediately or shortly after diagnosis due to bad prognoses or deterioration of the state of the animal. One dog with malignant peripheral nerve sheath tumor (case 8) received radiotherapy and had a survival of one year. Another case with suspected peripheral nerve sheath tumor (case 10) was lost on follow up. The ferret (case 4) was diagnosed with disseminated lymphoma and was euthanized after worsening of its clinical signs due to a suspected ascending myelomalacia.

## 4. Discussion

Percutaneous free-hand CT-guided FNB seems to be useful for the diagnosis of vertebral and paravertebral lesions in small animals. Through a correct interpretation of an adequate cytological sample, and a correlation with the clinical and imaging findings, a conclusive diagnosis can be made in most cases [13]. This may avoid the need for complementary TCB.

In the present study, a cytological diagnosis was obtained in 80% of the cases. A similar result was obtained in a study on bone lesions (83%) [7]. In our study, two FNB taken from a VB (case 11) and from an IVF (case 8) were non-diagnostic. Possible reasons for the non-diagnostic FNB are the difficult access to the lesions (in case 8, the lesion was located in the IVF and in case 11, in the interscapular region), causes inherent to the type of tumor (sarcomas and especially peripheral nerve sheath tumors exfoliate less than other type of tumors) or to the size of the lesion (in case 8, the lesion consisted of thickening of the nerves, without the presence of a mass). Other possible causes are the individual skills of the different operators and the possible rush associated to the time of anaesthesia of the patient itself. Furthermore, another possible reason for the unsuccessful FNB (case 9) was the poor visualization of the lesion after contrast medium was diluted with time.

Although the correlation of the neurological exam and the imaging diagnosis is high, biopsy remains the gold standard technique to reach the final diagnosis in vertebral lesions. Open biopsy, with an accuracy of 98% [14], is being replaced in human medicine by image-guided percutaneous TCB. Image-guided TCB has been proven as a viable, faster, more cost-effective procedure that reduces the possible complications associated to the surgery [15,16]. Reported accuracies for CT-guided TCB of vertebral lesions in human literature range from 83 to 96%. These results vary among studies and depending on the region of the vertebral column [15,17,18]. Biopsies taken from the thoracic column seem to be less accurate than biopsies taken from the lumbar column [19]. Biopsies of the cervical column are less commonly taken due to its difficulty, but also have shown high accuracies, around 95% [18,20]. In our study, the adequacy of the FNB technique seemed to depend more on the limiting factors explained above than on the region of the column. Besides, there was a small number of cases in each region of the column, and no conclusions can be drawn. Computed tomography-guided FNB of vertebral lesions is also used in human medicine especially in patients with a previous history of malignancy, to confirm or rule out vertebral metastases [21,22]. It is also used to detect infection and to differentiate primary from metastatic bone tumors in patients with no previous history of neoplasia [13,23]. It is a safe, less traumatic, rapid and relatively easy method in comparison with open biopsy or TCB [22,23], with accuracies close to 90% [24,25]. The main disadvantage of FNB versus TCB is that FNB only shows the cytological features, in comparison with TCB that shows the tissue architecture; thus the diagnostic power can be reduced. Therefore, it is important to be able to assess adequacy of the sample by on-site evaluation of the cytology [13,22]. The decision of taking a complementary TCB, when the FNB is insufficient, can help to reach a more reliable diagnosis. In our study, the on-site evaluation of the cytology was not always possible. Therefore, in the cases in which the FNB was not sufficient for the decision making process, a surgical biopsy was taken.

There is no study in veterinary literature that compares the accuracies of FNB and TCB in vertebral and paravertebral lesions. Both techniques have been compared in patients with general bone lesions and the reported accuracies were higher with TCB (100%) than with FNB (83%) [7]. In that study, three vertebral lesions were sampled with TCB and were diagnostic, whereas the only vertebral lesion sampled with FNB was not. However, the small number of patients with vertebral lesions prevents from drawing any conclusions about the accuracy of sampling this type of lesion.

In the present study, the size of the needle used was 22 G. Small needle sizes (22 G and 20 G) have been used in other studies in small animals and people because it prevents contamination of the sample with blood [7,22,25,26,27,28]. The nature of the lesions (size, shape and location) prevented from taking complementary TCBs in most cases. Lesions smaller than 3–4 cm cannot be sampled with common automated TCB needles, as the excursion of the needle is a limiting factor. In the human literature special biopsy needles are used to sample small vertebral lesions, like the Bonopty Insertion Set^®^ [16,17,18]. However, in a comparative study, these needles were rated as being difficult to use and having the lowest yield [29].

Complications (worsening of the lameness and increased pain) were detected only in one dog in this study (case 2, severe discospondylitis). It is not clear whether these complications were due to the severe osteolysis and the expected progression of the infection after diagnosis, or to the FNB and TCB procedure. The low rate of complications (9%) agrees with the human literature, where complications after spinal CT-guided percutaneous sampling are in the range of 0–10% and less frequent than after surgical procedures [15,27].

One of the limitations of this study is its retrospective nature. The time required for the procedure was not recorded, but it was usually approximately one hour. Operator skills are important to reduce these timings which mainly affect the time of anaesthesia. In a previous study similar timings were reported for a CT-guided vertebral biopsy, which lasted 60 min approximately, in comparison with the shorter times needed for a nasal biopsy (20 min) [7]. A more specific diagnosis might have been reached in some cases (cases 1, 3, 6, 10, 11) with the use of TCB, which was only done in one case. In most cases only one or two samples were taken from the lesion, reducing the chances of reaching a diagnosis. Nevertheless, most of the samples were of good quality and diagnostic. Other limitations of this study were the small number of animals and the lack of obtaining a final diagnosis with necropsy and histopathology or surgical biopsy in some cases.

Fluoroscopy and ultrasound are other imaging modalities used to guide the FNB or TCB procedures [28]. Some lesions are not accessible to ultrasonographic evaluation, being vertebral lesions the most difficult to evaluate [1]. Therefore, ultrasound-guided sampling of the vertebral column appears to be a less indicated method than CT to this purpose. On the other hand, fluoroscopy-guidance has the disadvantages of having a lower contrast resolution than CT and not being free of superimposition, making visualization of paravertebral lesions difficult. Furthermore, the physician will be exposed to ionizing radiation. Nevertheless, both ultrasound-guided and fluoroscopy-guided percutaneous FNB of the intervertebral disc have been reported in dogs with discospondylitis [30,31]. The advantages of CT over ultrasound and fluoroscopy have already been explained. The main disadvantage of CT is that real-time imaging is not possible with the common equipment used in veterinary medicine. This could lengthen the procedure time and increase the risk of needle slippage into adjacent structures [15]. In a meta-analysis in people, CT and fluoroscopic guidance resulted in similar accuracies and rate of complications [15]. Therefore, the choice for the guidance modality will depend on factors like the preference of the physician or the location of the lesion.

In conclusion, CT-guided FNB of vertebral and paravertebral lesions appears to be a safe and useful technique with minor complications in small animals. Nevertheless, a degree of expertise of the physician is important to obtain adequate samples and reduce the time of the procedure.

## Figures and Tables

**Table 1:** Results of the free-hand CT-guided fine needle biopsies (FNB) in 10 dogs and 1 ferret.

Case	Species Breed Gender Age	History and Clinical Signs	Neurological Examination (NE) and Neurolocalization (NLoc)	CT Findings Suspected Imaging Diagnosis	Location of Tip of the FNB Needle	Diagnosis from CT-Guided FNB (Cytology/Culture)	Diagnosis from Surgical Biopsy or Necropsy and Histopathology
1	Canine German shepherd dog Male intact 7 yo	Chronic progressive hindlimb weakness	NE: absence of postural reactions (PR) in both HLs, normal spinal reflexes (SR) and thoracolumbar hyperesthesia NLoc: T3-L3 myelopathy	Polyostotic aggressive bone lesion (mainly lytic) in L3 and L4 articular processes, laminae and pedicles, mostly right-sided. Non-primary bone tumor, synovial cell sarcoma	Right L4 cranial articular process	Mesenchymal tumor consistent with sarcoma	
2	Canine Staffordshire bull terrier Male intact 6 yo	Chronic progressive lumbosacral pain (currently severe pain)	NE: No neurological deficits associated NLoc: LS region	Aggressive lesion centered in the IVDS at L7-S1. Severe endplate osteolysis, moderate sclerosis, narrowing of the IVDS and marked NBF. Discospondylitis (chronic)	Intervertebral disc L7-S1	Discospondylitis by *Enterobacter cloacae*	
3	Canine Bodeguero andaluz Female intact 9 yo	Chronic, progressive, intermittent RHL lameness.	NE: Currently LMN monoparesis of the RHL. Absence of PR in the RHL, decreased withdrawal reflex. NLoc: L6-S2 spinal segment, L6-S2 right nerve roots or L6-S2 right spinal nerves.	Intradural extramedullary right-sided tubular mass at the level of L5 VB and L5–L6 IVF. Peripheral nerve sheath tumor	Right IVF L5–L6	Mesenchymal tumor consistent with sarcoma	
4	Ferret Female neutered 6 yo	Acute onset of paraplegia	NE: Absence of PR and nociception and decreased withdrawal reflex in both HLs. CTMR cut off L3. TL hyperesthesia. NLoc: T3-L3 myelopathy and associated spinal shock	Polyostotic aggressive bone lesions (mainly lytic) in vertebrae and ribs, worst on L1. Non-primary bone tumor	Right L1 VB and TP	Lymphoma	Disseminated lymphoma (necropsy)
5	Canine Labrador retriever Male intact 2 yo	Chronic progressive HLs weakness and ataxia.	NE: ambulatory paraparesis and proprioceptive ataxia of the HLs. Paresis of the tail. Decreased muscle tone of HLs and tail. Absent PR and SR in the HLs and decreased perineal reflex. NLoc: L4-Cd5 myelopathy +/− L4-Cd5 nerve roots +/− L4-Cd5 spinal nerves	Severe thickening/mass located in the epidural space in the vertebral canal along L5–L7, expanding to the L5–L6 IVF (R > L). Infiltrative neoplasia (lymphoma, sarcoma)	Right L5–L6 IVF	Lymphoma	
6	Canine Weimaraner Female intact 13 yo	Chronic progressive weakness and incoordination of the HLs and spinal pain	NE: Ambulatory paraparesis. Absence of PR and normal SR in the HLs. TL hyperesthesia. NLoc: T3-L3 myelopathy	Large mass in right hypaxial muscles infiltrating VB and TP of L3 and L4 and retroperitoneal space (CVC). Pathological fracture of L3 VB with vertebral compression. Lung nodules and periaortic lymphadenopathy. Soft tissue sarcoma	Right L3 VB/ TP and hypaxial mass	Mesenchymal tumor consistent with sarcoma	
7	Canine Cross breed large Female neutered 10 yo	Chronic and progressive history of severe cervical hyperesthesia, TL kyphosis	NE: Ambulatory tetraparesis worst in the HLs; absence of PR with normal SR in all four limbs. Severe hyperesthesia in the C- and TL- spine. NLoc: multifocal C1–C5 and T3-L3 myelopathy	Multifocal aggressive lesions centered in the IVDS of the C- and T- spine, worst at C6–C7 and T11–T12. Subluxation at C6–C7. Severe endplate osteolysis, sclerosis and narrowing of the IVDS. Marked NBF. Discospondylitis (chronic)	IVDS at T11–T12	Discospondylitis by *Pseudomona aeruginosa*	-
8	Canine Fox terrier Male intact 9 yo	Chronic and progressive lameness of the RHL	NE: monoparesis of the RHL, mildly decreased muscle tone. Decreased PR and SR in RHL. Mild discomfort on palpation of the LS vertebral column. NLoc: L6–L7 spinal cord segments, L6–L7 nerve roots or L6–L7 spinal nerves (right side)	Moderate thickening of several nerve roots and spinal nerves (L5–L6 right, L6–L7 right and L7-S1 bilaterally). Moderate right-sided muscle atrophy. Peripheral nerve sheath tumor	Right L6-7 IVF	Not diagnostic	Benign peripheral nerve sheath tumor (surgical biopsy)
9	Canine West Highland white terrier Female intact 10 yo	Intermittent left-sided circling, bilateral HLs weakness, progressing to FLs weakness	NE: Proprioceptive ataxia and paraparesia in HLs. Left vestibular ataxia. Absent PR with normal SR. Slight discomfort on palpation of the C- spine. NLoc: C1–C5 myelopathy	Intradural extramedullary mass corresponding to the left ventral spinal root of C2, severely compressing and invading the spinal cord, and extending extra-axially as a large tubular mass with strong perineural enhancement. Severe left-sided paraspinal muscle atrophy. Peripheral nerve sheath tumor	Tip of the needle out of the lesion.	Not evaluable	Malignant peripheral nerve sheath tumor (necropsy)
10	Canine Galgo español Female intact 12 yo	Acute bilateral HLs weakness	NE: Non ambulatory paraparesis, absent PR with normal SR in the HLs. Present crossed extensor reflex in both HLs. NLoc: T3-L3 myelopathy	Large right-sided intradural extramedullary mass at T8, severely compressing the spinal cord and extending extra-axially through the spinal nerves from T6 to T9. Peripheral nerve sheath tumor	Right T8–T9 IVF (thickened nerve)	Mesenchymal tumor, consistent with sarcoma	-
11	Canine Crossbreed Male intact 9 yo	Chronic neck pain. Acute right FL tremors and lameness	NE: Ambulatory tetraparesis. Reduced PR with normal SP in all 4 limbs. Severe hyperesthesia in the C-spine NLoc: C1–C5 myelopathy	Monostotic aggressive bone lesion in C6 (osteolytic and osteoproductive), mainly right-sided, with moderate extradural compression of the spinal cord. Primary bone tumor	Right C6 cranial articular process.	Not diagnostic	Osteosarcoma (surgical biopsy)

C: cervical; CMTR: Cutaneous trunci muscle reflex; CT: computed tomography; CVC: caudal vena cava; FLs: front limbs; FNB: fine needle biopsy; HLs: hind limbs; IVDS: intervertebral disk space; IVF: intervertebral foramen; L: lumbar; LMN: low motoneuron; LS: lumbosacral; NBF: new bone formation; NE: neurological exam; NLoc: neurolocalization; PR: postural reactions; RHL: right hindlimb; SR: spinal reflexes; T: thoracic; TL: thoracolumbar; TP: transverse process; VB: vertebral body; yo: years old.

## Data Availability

The data presented in this study are available on request from the corresponding author.
